# Adult attachment and emotion dysregulation in borderline personality and somatoform disorders

**DOI:** 10.1186/s40479-015-0026-9

**Published:** 2015-03-28

**Authors:** Annemiek van Dijke, Julian D Ford

**Affiliations:** VU University Amsterdam Faculty of Psychology and Education Van der Boechorststraat, 1 NL- 1081 BT Amsterdam, Netherlands; Department of Psychiatry, University of Connecticut Health Center, Farmington, CT USA

**Keywords:** Borderline personality disorder, Somatoform disorder, Attachment, Emotion regulation, Alexithymia

## Abstract

**Background:**

Borderline personality disorder (BPD) and somatoform disorders (SoD) involve significant problems in relationships and emotion regulation, but the similarities and differences between these disorders in these areas is not well understood.

**Method:**

In 472 psychotherapy inpatients BPD and/or SoD diagnoses were confirmed or ruled out using clinical interviews and standardized measures. Emotional under- and over-regulation and indices of adult attachment working models and fears were assessed with validated self-report measures. Bivariate and multivariate analyses were conducted to examine relationships among the study variables and differences based on diagnostic status.

**Results:**

Under-regulation of emotion was moderately related to fear of abandonment but weakly related to fear of closeness. Over-regulation of emotion was moderately related to fear of closeness but not to fear of abandonment. BPD was associated with under-regulation of emotion and fear of abandonment, and, when comorbid with SoD, with fear of closeness. SoD was associated with inhibition or denial of fears of abandonment or closeness, and over-regulation of emotion.

**Conclusions:**

Study results suggest that insecure attachment may play a role in both BPD and SoD, but in different ways, with hyperactivating emotion dysregulation prominent in BPD and deactivating emotion dysregulation evident in SoD. Also, combined hyper- and de-activating strategy components that may reflect a pattern of disorganized attachment were found, particularly in patients with comorbid BPD and SoD.

## Background

Attachment theory has become a prominent conceptual framework for understanding the process of emotion regulation and dysregulation. Bowlby ([1-4]) highlighted the anxiety-buffering and physical protection functions of close relationships and conceptualized proximity-seeking as an emotionally regulated alternative to the instinctive and typically dysregulated fight-flight responses. He also emphasized the importance of interpersonal experiences as sources of individual differences in emotion regulation over one’s lifetime. Elaborating on Bowlby’s work, Mikulincer and colleagues proposed a model of activation and dynamics (hyperactivation versus deactivation) of the attachment system. [5,6]. Mikulincer’s model hypothesizes that, when confronted with potential threatening events, the *primary attachment strategy* involves proximity seeking: attempting to move closer, physically or emotionally or both, to persons who are perceived as providing relational security that can serve to alleviate distress and build or access resources.

When external (real) or internalized (i.e., working model representations of) attachment figures are unavailable, *secondary attachment strategies* (hyperactivation or deactivation of the internalized attachment system) are hypothesized to be utilized in order to cope with relational insecurity and related distress*.* Secondary attachment strategies involve a defensive focus either on fear of abandonment (i.e., attempts to restore proximity and reduce anxiety; hence hyperactivation) or fear of closeness (i.e., attempts to inhibit proximity seeking and reduce awareness of distress; hence deactivation). Clinically and phenomenologically, the secondary attachment strategies appear to involve relatively distinct forms of emotion dysregulation, with under-regulation of emotion predominating in fear of abandonment and over-regulation of emotion characterizing fear of closeness.

Secondary attachment strategies may be of particular relevance to the kinds of dysfunctional self- and emotion regulation, especially in an interpersonal context, that are observed clinically in patients with mental disorders and more specifically in borderline personality disorder (BPD) and somatization disorders (SoD). Whereas both diagnoses are associated with difficulties with relationships and intimacy, they appear to involve distinct forms of secondary attachment strategies. BPD has been associated with secondary attachment strategies involving under-regulation of emotion, while SoD is associated with secondary attachment strategies involving over-regulation of emotion [7-11]. However, the role that adult attachment fears, and hyper-activation or deactivation of the internalized attachment system, plays in these forms of emotion dysregulation in BPD and SoD remains unclear and is therefore the focus of the present study.

Only one study has assessed both emotion dysregulation and adult attachment in patients with SoD. Results from that investigation showed that dismissing attachment working models (associated with fear of closeness) were common in this clinical sample and associated with alexithymia and over-regulation of emotion [12].

In the empirical and clinical literature, across a variety of measures and attachment typologies, two variants of fear of abandonment (i.e., preoccupied and fearful attachment) have been reported as characterizing BPD (e.g., [13]). BPD is consistently found to be specifically associated with both emotion dysregulation and an extreme fear of interpersonal rejection [14]. However, no studies have simultaneously examined adult attachment strategies and emotion dysregulation in BPD.

Therefore, this study attempted to determine whether BPD with or without SoD was associated with specific combinations of emotion dysregulation and adult attachment working models. It was hypothesized that: (1) fear of abandonment and dysfunctional under-regulation of emotion (hyperactivation of the internalized attachment system) will be associated with BPD, compared to SoD or other mental disorders, (2) fear of closeness and dysfunctional over-regulation of emotion (deactivation of the internalized attachment system) will be associated with SoD compared to BPD or other mental disorders.

## Methods

### Design, setting and participants

In total 472 participants diagnosed with BPD only, SoD only, BPD with co-morbid SoD or other mental disorder (i.e. depression or anxiety disorder) as psychiatric comparisons participated in the multi-center psychotherapy project “Clinical Assessment of Trauma-Related Self and Affect Dysregulation” [7]. Approximately one-third met criteria for SoD only, 25% for BPD only, and 25% for comorbid BPD + SoD, and 17% for depression or anxiety disorders with neither SoD nor BPD. Demographic characteristics of the four study groups are presented in Table 1. Briefly, the sample was more than two-thirds female, primarily young and mid-life adults, half involved in a primary couple relationship, and a mix of education levels ranging from not graduating from secondary-level school to secondary school graduation to some college-level education. No significant effects were found in the full sample for sex, age, primary relationship status, or level of education on the dependent variables, therefore demographic variables were not used as covariates in subsequent analyses.

**Table 1:** **Demographic Characteristics of BPD, SoD, BPD + SoD, PC Sub-groups and the Total Sample**

	**BPD**	**SoD**	**BPD + SoD**	**PC**	**Total Sample**
**N** =	120	159	129	64	472
Male	40	47	30	28	145
Female	80	112	99	36	327
**Age** M (SD)	29.9 (8.8)	38.3 (10.5)	33.6 (9.1)	36.8 (9.9)	34.7 (10.1)
**Social** N	30.8%	45.3%	40.3%	28.1%	37.9%
T	60.8%	41.5%	47.3%	56.3%	50.0%
S	8.3%	13.2%	12.4%	15.6%	12.1%
**Education** L	24.2%	22.6%	27.1%	23.4%	24.4%
M	35.8%	45.9%	37.2%	46.9%	41.1%
H	40%	31.4%	35.7%	29.7%	34.5%

Note: BPD, borderline personality disorder; SoD, somatoform disorder; BPD + SoD borderline personality disorder and somatoform disorder; PC, psychiatric comparison group; N, no primary partner; T, living together; S, separated by death or divorce; Education, highest level of education attained; L, primary or some secondary education; M, completed secondary education; H, college-level education.

Diagnoses of BPD and SoD were made according to the DSM-IV criteria during intake by certified clinicians (psychiatrists, psychotherapists). Where possible, general practice and former hospital records were obtained (with patient’s consent) and studied. All participants had a well-documented history of somatic and/or psychiatric symptoms. All had received previous inpatient or outpatient treatment at psychiatric or medical hospitals and were referred for specialized tertiary treatment. Also, DSM-IV BPD and SoD (i.e., somatization disorder, undifferentiated somatoform disorder, severe conversion and pain disorder) diagnoses were confirmed by trained clinicians using structured clinical interviews (under supervision AvD).

The *Composite International Diagnostic Interview* (CIDI- section C for somatoform and dissociative disorders; World Health Organization, [15,16]; Dutch version Ter Smitten, Smeets, & Van den Brink, [17]) is a comprehensive, standardized instrument for assessing mental disorders according to the definitions and diagnostic criteria of DSM-IV and ICD-10. Moreover, the diagnoses of SoD or other (depression or anxiety) disorders was confirmed or ruled out by a psychiatrist with experience with psychosomatic disorders, a specialist in internal medicine, or a general practitioner with psychiatric experience.

The *Borderline Personality Disorder Severity Index* (BPDSI; [18]; Dutch version IV, [19]) is a semi-structured interview that contains nine sections (abandonment, relationships, self-image, impulsivity, parasuicide, emotion, emptiness, anger, and dissociation and paranoia) corresponding to the nine symptom clusters of BPD. Each section contains items asking about incidents in which a symptom occurred, for example, “Did you, during the last three months, ever become desperate when you thought that someone you cared for was going to leave you?” The items are scored by the interviewer using a 10-point scale, indicating how often the event happened during the last three months. An average score was calculated for each section; total scores were calculated by summing the section scores. The BPDSI has been shown to have good validity and reliability [20]. In addition to meeting DSM-IV criteria, severity cut-off score of 20 on the BPDSI-total score was used (personal communication, Arntz, October 2003) for inclusion in the study.

This study was approved by the local medical ethics committee for mental health research (METiGG). All subjects provided written informed consent to participate after the procedure had been fully explained, according to the Declaration of Helsinki.

### Measures

In order to assess under-regulation of emotion, each subject completed a sub-scale from the Dutch self-report version of the *Structured Interview for Disorders of Extreme Stress Not Otherwise Specified, Revised* (SIDES-rev; [21]). The SIDES-rev-NL is an adaptation of the SIDES-rev interview which provides a sub-scale for dysregulated emotion ([21]; Dutch translation and back-translation by [22]). The criterion for presence of pathological under-regulation of emotion was adopted from the SIDES-rev scoring manual [21]: Criterion I.a. “emotion dysregulation” requires that 2 out of 3 items are answered ≥ 2, on a 0–3 scale, where “2” represents clinically significant symptoms and “3” indicates extremely severe symptoms). The three Criterion I.a. items include: (1) often getting “quite upset over daily matters, (2) being unable to get over the upset for hours or not being able to stop thinking about it, and (3) having to “stop everything to calm down and it took all your energy” or “getting drunk, using drugs or harming yourself” to cope with emotional distress. Thus, the measure addresses the core components of under-regulation of emotion, i.e., frequent/intense distress, inability to modulate or recover from distress, and use of self-defeating coping to deal with distress. The SIDES self-report-NL version has not been validated in a BPD or SoD population; therefore, we performed reliability analysis and found that the emotion dysregulation sub-scale was reliable in this sample (Cronbach’s Alpha = .75). Evidence of convergent and discriminant validity of the sub-scale in the BPD and SoD samples in this study was found in relation to independent measures of excitatory and inhibitory experiencing [10] and under- and over-regulation of emotion [23], and construct validity was supported in relation to a measure of childhood history of traumatic experiences with primary caretakers [9,11].

In order to assess ‘over-regulation of emotion’, each subject completed the *Bermond Vorst Alexithymia Questionnaire* (BVAQ; [24]), which is a Dutch forty-item questionnaire with good psychometric qualities [24], encapsulating two distinct second order factor groupings: cognitive dimensions (inhibited verbalizing, identifying, and analyzing emotions) and emotionive dimensions (inhibited emotionalizing and fantasizing). High scores represent stronger alexithymic tendencies: “diminished ability to”… The reliability for the total scale and its subscales is good and varies between 0.75 and 0.85 [24]. A reliability analysis was performed for the whole sample and the BVAQ proved to be reliable (Cronbach’s 〈 = 0.88). The cognitive factor of the BVAQ was used to assess over-regulation in order to enable comparisons with previous studies [12,25]. The cognitive factor of the BVAQ is highly correlated with the Toronto Alexithymia Scale (TAS-20; [26]; r = 0.80).

Adult attachment was assessed using the Dutch version of the *Relationship Style Questionnaire* (RSQ; Griffin & Bartholomew, [27,28]). The RSQ was translated into Dutch and retranslated by a near-native speaker and with permission of the authors (KB). The RSQ is a 30-item questionnaire that asks about feelings, thoughts, and behaviors in relationships. The RSQ measures categorical and dimensional aspects of adult attachment. The four-category model consists of secure, dismissing, preoccupied, and fearful adult attachment styles. The RSQ has demonstrated good reliability and convergent validity (Bartholomew & Horowitz, [29]). Following Steffanowski et al. [30], dimensional scores were derived for fear of abandonment (attachment anxiety) and fear of closeness (attachment avoidance), while categorical classifications were computed by applying cut-off scores for fear of abandonment (2.87) and fear of closeness (2.75).

### Statistical analysis

All analyses were performed using SPSS, version 16 (SPSS Chicago). Relationships between the measures of affect dysregulation and RSQ scores for fear of abandonment and fear of closeness were evaluated using Pearson correlations (using two-tailed tests). Group means for the continuous RSQ scores were compared using multivariate analyses of variance (MANOVAs) with diagnosis as independent variable. Sequential regression analyses were conducted. The following contrasts were tested: (1) PC (Psychiatric Comparisons not meeting criteria for BPD and/or SoD) versus all participants diagnosed with BPD and/ or SoD, (2) BPD versus SoD, (3) BPD + SoD versus BPD, and (4) BPD + SoD versus SoD. Under-regulation and over-regulation were entered first into each regression analysis (model 1), and followed by adding adult attachment styles (RSQ categorical sub-groups: secure, dismissive, fearful, and pre-occupied; model 2), and finally adding the attachment dimensions of fear of abandonment and fear of closeness (model 3). Cross tabulations with chi square tests and standard residual values were used to determine whether the RSQ categorical sub-groups were represented differentially among the diagnostic groups. Standard residuals are a way of contrast testing. Standard residual values less than −2 or greater than 2 represent statistically meaningful separation between groups. A negative value denoted “less frequent than expected”; a positive value denoted “more frequent than expected” compared to all other groups.

## Results

When considering the sample as a whole, under-regulation was moderately related to fear of abandonment (attachment anxiety; r = 0.30, *p* < 0.001) and weakly related to fear of closeness (attachment avoidance; r = 0.16, *p* < 0.001). Over-regulation was moderately related to fear of closeness (r = 0.44, *p* < 0.001) but not to fear of abandonment (r = −0.03, *p* > 0.05). Under-regulation and over-regulation were weakly related (r = 0.11, *p* < 0.02). Fear of abandonment and fear of closeness were not related (r = 0.08, *p* > 0.05).

Also, under-regulation was moderately related to fearful (r = 0.30, *p* < 0.001) and weakly related to secure (r = −0.15, *p* < 0.001), dismissing (r = 0.09, *p* < 0.05), and preoccupied (r = 0.17, *p* < 0.001) adult attachment scores. Over-regulation was moderately related to fearful (r = 0.30, *p* < 0.001) and weakly related to secure (r = −0.12, *p* < 0.01), and dismissing (r = 0.11, *p* < 0.02) adult attachment scores, but was not related to preoccupied (r = −0.07, *p* < 0.11) adult attachment sores.

The MANOVA exploring group differences in deactivating and hyperactivating strategy components (adult attachment scores, attachment fears, and affect dysregulation) revealed a statistically significant difference between diagnostic groups: *F* (24, 1329) = 10.62; *p* = 0.001; Wilks’ Lambda = 0.60; partial eta squared = 0.16). When the results for the dependent variables were considered separately, between-group differences were found for all dependent variables (Table 2). Medium effect sizes were found for between group differences on secure- and fearful- adult attachment, fear of abandonment, and under-regulation of affect. Small effect sizes were found for dismissing and preoccupied adult attachment, fear of closeness, and over-regulation of affect.

**Table 2:** **Multivariate Analysis of Variance (MANOVA) of Between Group Differences (BPD vs. SoD vs. BPD + SoD vs. PC) for Adult Attachment Fears and Affect Dysregulation**

	**F (3, 464)**	**Partial eta-squared**
Secure adult attachment fears	26.5***	.15
Dismissing adult attachment fears	8.82***	.05
Fearful adult attachment fears	27.22***	.15
Pre-occupied adult attachment fears	11.23***	.07
Under-regulation of affect	26.31***	.15
Over-regulation of affect	4.89**	.03
Fear of Abandonment	25.78***	.14
Fear of Closeness	14.44***	.09

Note: BPD, borderline personality disorder; SoD, somatoform disorder; BPD + SoD, borderline personality disorder and somatoform disorder; PC, psychiatric comparison group.

*p ≤ .05; **p ≤ .01; ***p ≤ .001.

Table 3 presents the means and standard deviations of adult attachment and fear of abandonment or closeness for all study groups. The mean scores reflect the overall finding that BPD (with or without SoD) was associated with high levels of fearful attachment, fear of abandonment, and fear of closeness, and low levels of secure attachment compared to both the SoD and PC groups. Similar but relatively smaller elevations can be observed for BPD (with or without SoD) with regard to preoccupied attachment. Both BPD and SoD were associated with higher levels of dismissive attachment, compared to the PC group. Post hoc comparisons with PC as reference group, revealed that the SoD group was more likely to report deactivating secondary attachment strategies (dismissing attachment in combination with over-regulation), whereas the BPD group was more likely to report hyperactivating secondary attachment strategies (preoccupied adult attachment in combination with under-regulation). The BPD + SoD group was more likely to report a combination of both hyperactivating and deactivating secondary attachment strategies (both under and over-regulation of affect and fearful adult attachment).

**Table 3:** **Means (Standard Deviations) for Adult Attachment Scores, Attachment Fears, and Affect Dysregulation by Diagnostic Group with post-hoc comparisons**

**Group**	**N**	**Secure**	**Fearful**	**Dismissive**	**Preoccupied**	**Fear of Abandonment**	**Fear of Closeness**	**Under-regulation of affect**	**Over-regulation of affect**
BPD									
	119	2.36^a^	3.84c	3.56c	3.22c	3.20c	3.51c	8.29c	77.06c
		(.63)	(.78)	(.70)	(.85)	(.68)	(.81)	(1.84)	(17.89)
SoD									
	159	2.65^b^	3.23a	3.59c	2.74b	2.56a	3.04a	6.64b	72.63b
		(.63)	(.90)	(.67)	(.78)	(.73)	(.79)	(2.02)	(17.54)
BPD + SoD									
	129	2.29^a^	4.00c	3.73c	3.12c	3.10c	3.58c	8.44c	79.26c
		(.61)	(.66)	(.62)	(.87)	(.71)	(.72)	(1.83)	(17.90)
PC									
	62	3.10^c^	3.41b	3.19b	2.74b	2.68b	3.26b	7.19b	70.70b
		(.81)	(.78)	(.77)	(.67)	(.61)	(.80)	(2.30)	(19.55)

Note: BPD, borderline personality disorder; SoD, somatoform disorder; BPD + SoD, borderline personality disorder and somatoform disorder; PC, psychiatric comparison group. Groups with different superscripts differed *p* < .05 on *post hoc* comparisons with PC as reference group.

Sequential regression analyses results are presented in Table 4. For all contrasts except for BPD versus BPD + SoD, the inclusion of all deactivating and hyperactivating experiencing phenomena (model 3) improved the fit of the model significantly (PC↔the rest: Cηι^2^ = 70.51, *df* = 8*, p* < 0.000; BPD↔SoD: Xηι^2^ = 87.94, *df* = 8*, p* < 0.000; BPD↔BPD + SoD: Xηι^2^ = 8.33, *df* = 8*, p* < 0.40; SoD↔BPD + SoD: Xηι^2^ = 111.09, *df* = 8*, p* < 0.000). No significant differences were found for deactivating and hyperactivating secondary attachment strategies between the BPD group and the BPD + SoD group. The Hosmer-Lemeshow test revealed that, for all dependent variables, model 2 fits the data well (PC↔the rest: Xηι^2^ = 9.72 *df* = 8, *p* = 0.29; BPD↔SoD: Xηι^2^ = 8.61, *df* = 8, *p* = 0.38; BPD↔BPD + SoD: Xηι^2^ = 6.28, *df* = 8, *p* = 0.62; SoD↔BPD + SoD: Xηι^2^ = 7.23, *df* = 8, *p* = 0.51). Compared to the SoD group, the BPD group was more likely to report under-regulation of affect and fear of abandonment. The SoD group was more likely to deny under-regulation of affect, fear of abandonment, and fear of closeness than the BPD + SoD group.

**Table 4:** **Results of Sequential Regression Analyses for deactivating and hyperactivating experiencing phenomena using contrast testing**

**N = 472**	**Odds Ratio**	**95.0% C.I. For Odds Ratio**	
**PC versus participants diagnosed with BPD and/or SoD**			
Over-regulation of affect	.98**	.96	1.00
Under-regulation of affect	.97	.84	1.12
Secure adult attachment fears	3.66***	2.30	5.83
Fearful adult attachment fears	.99	.59	1.67
Dismissive adult attachment fears	.43***	.27	.68
Preoccupied adult attachment fears	.79	.46	1.33
Fear of abandonment	.87	.49	1.56
Fear of closeness	1.84*	1.05	3.23
**BPD versus SoD**			
Over-regulation of affect	1.00	.99	1.02
Under-regulation of affect	1.37***	1.17	1.62
Secure adult attachment fears	.94	.55	1.59
Fearful adult attachment fears	1.30	.76	2.22
Dismissive adult attachment fears	.78	.48	1.27
Preoccupied adult attachment fears	1.07	.67	1.71
Fear of abandonment	2.36**	1.39	4.02
Fear of closeness	1.62	.91	2.91
**BPD versus BPD + SoD**			
Over-regulation of affect	.99	.98	1.01
Under-regulation of affect	.97	.85	1.12
Secure adult attachment fears	1.29	.79	2.10
Fearful adult attachment fears	.67	.39	1.13
Dismissive adult attachment fears	.79	.50	1.25
Preoccupied adult attachment fears	.96	.63	1.48
Fear of abandonment	1.30	.77	2.19
Fear of closeness	1.46	.84	2.53
**SoD versus BPD + SoD**			
Over-regulation of affect	.99	.97	1.01
Under-regulation of affect	.67***	.56	.79
Secure adult attachment fears	1.34	.79	2.30
Fearful adult attachment fears	.54*	.31	.93
Dismissive adult attachment fears	1.07	.65	1.78
Preoccupied adult attachment fears	.89	.55	1.44
Fear of abandonment	.55*	.33	.93
Fear of closeness	.68	.38	1.20

Note: *p < 0.05; **p < 0.01; ***p < 0.001; BPD, borderline personality disorder group; SoD, somatoform disorder group; BPD + SoD, borderline personality disorder and somatoform disorder group; PC, psychiatric comparison group; italic numbers indicate inverse relations.

**Figure 1:**
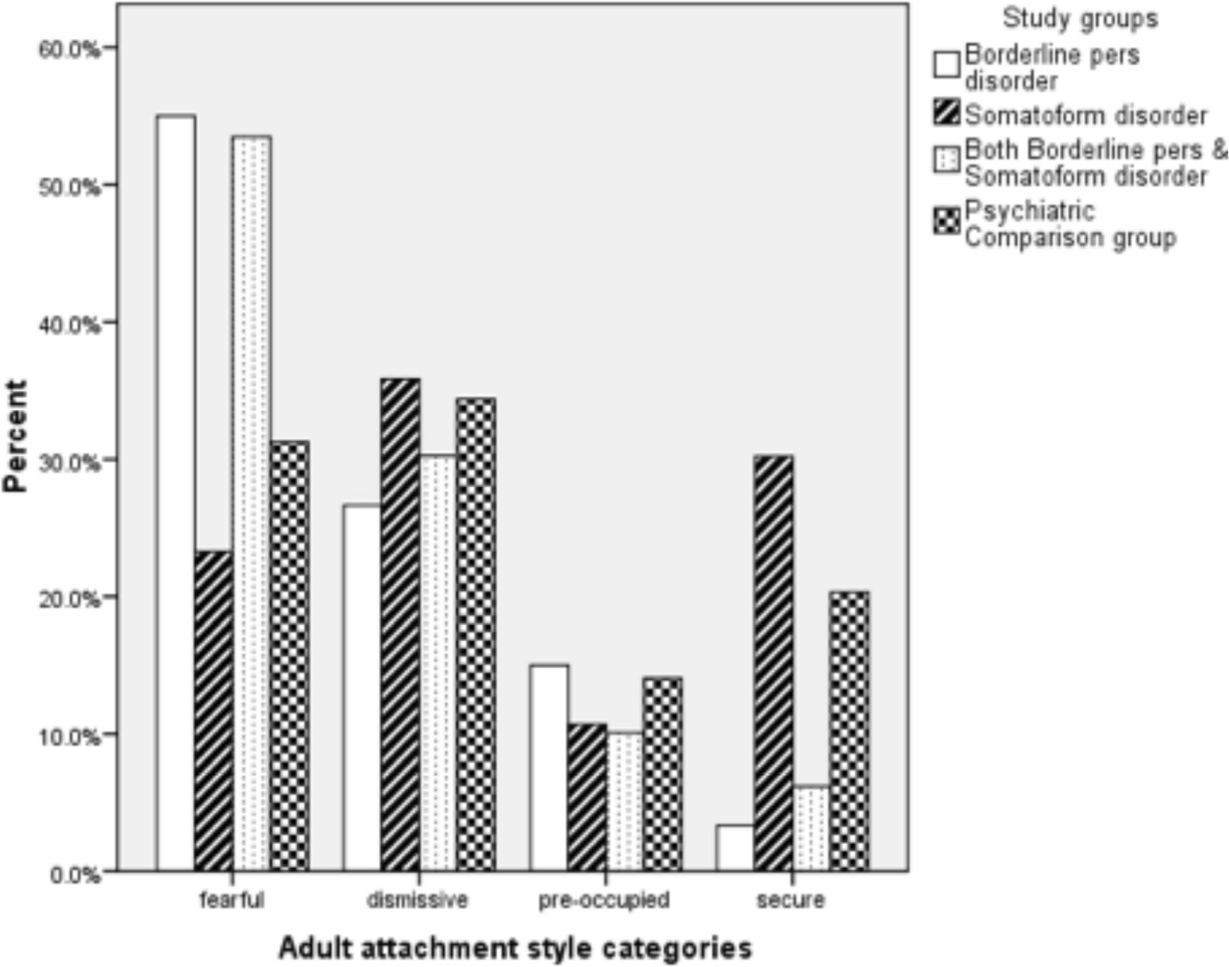
**Distribution of study groups for adult attachment styles.**

Using the described cut-off scores, 15.5% of the sample reported secure adult attachment, 12.1% reported preoccupied, 31.8% reported dismissing, and 40.7% reported fearful adult attachment. Figure 1 presents results about adult attachment for the BPD, SoD, BPD + SoD, and psychiatric comparison groups. Significant group differences were found (Xηι^2^ = 70.12, df = 9, *p* < 0.000). The SoD group was significantly more likely to report secure adult attachment (standardized residual value (SRV) = 4.7), and less likely to report fearful adult attachment (SRV = −3.4) than were the BPD and BPD + SoD groups. Fearful adult attachment was significantly more frequently reported by participants with BPD (SRV = 2.5) or BPD + SoD (SRV = 2.3) diagnoses, while secure adult attachment was less frequently reported by BPD (SRV = −3.4) and BPD + SoD (SRV = −2.7), than by the SoD or PC groups.

## Discussion

Consistent with study hypotheses, psychometric assessments with adult psychiatric patients revealed distinct patterns of secondary attachment strategies and emotion dysregulation associated with BPD and SoD. Patients with SoD alone tended to deny fearful secondary attachment strategies and to endorse dismissive attachment strategies and over-regulation of emotion, consistent with a deactivating approach to attachment and emotion regulation. These findings are consistent with prior studies showing SoD to be related to a temperament style of low effortful control [31] and agreeableness [32] and, defensive engagement of the behavioral inhibition system [33]. In contrast, BPD was associated with both under-regulated emotion and fearful secondary attachment strategies specifically related to abandonment, consistent with a hyperactivating approach to attachment and emotion regulation. These findings are consistent with evidence linking BPD with a negative emotionality temperament style [31], heightened engagement of the behavioral activation system [33], extreme fear of rejection [14], and bio-affective dysregulation [34].

The results of this study suggest that, although both BPD and SoD are often associated with insecure attachment [12,13,25], specific secondary attachment strategies of fear of abandonment (hyperactivation of the attachment system) or closeness (deactivation of the attachment system) appear to differentiate these clinical conditions. In addition to under-regulation of emotion, fear of abandonment was particularly associated with BPD.

However, contrary to study hypotheses, fear of closeness also was associated with BPD when it co-occurred with SoD. Strikingly, SoD, while associated with over-regulation of emotion, was not consistently associated with reports secondary attachment strategies except a dismissive style, unless SoD was comorbid with BPD. When SoD occurred without BPD, fear of abandonment was particularly uncommon whereas fear of closeness was more often reported. The latter finding is consistent with study hypotheses and with prior research on insecure attachment in SoD [12,25].

Consistent with study hypothesis, fear of abandonment and fear of closeness appeared to be distinct phenomena. Fear of abandonment was most closely associated with under-regulated emotion and BPD, while fear of closeness was most strongly associated with over-regulated emotion and SoD. In addition, the combination of both under- and over-regulation of emotion and fears of closeness and abandonment were most strongly associated with comorbid BPD + SoD [7,10,23,35]. This combination of typically divergent secondary attachment styles and forms of emotion dysregulation is consistent with a disorganized/ disoriented attachment style [36] and potentially adds a third form of activation to Mikulincer and Shaver’s model of activation of the attachment system.

These findings may enhance clinical practice by suggesting that when assessing SoD it is important to consider denial of emotional experiencing and attachment fears (low scores), as well as reports of dismissing attachment and difficulties differentiating emotions/alexithymia. Whereas for BPD, although hyperactivation of the attachment system and under-regulation of emotion are likely to be evident, there may be a sub-group who also experience somatoform symptoms and deactivation of the secondary attachment system (i.e., fear of closeness as well as of abandonment; over- as well as under-regulation of emotion).

Consequently, treatment protocols for emotion regulation problems in BPD and SoD should be designed to address both under- and over-regulation as well as the full range of secondary attachment strategies for those diagnosed with BPD + SoD. This comorbid sub-group is likely to report symptoms associated with both types of emotion dysregulation and both hyperactivation and deactivation of the attachment system, which may be consistent with the presence of complex PTSD and PTSD dissociative subtype [14]. Indeed, complex PTSD was reported more frequently in this comorbid BPD + SoD subgroup than in the other groups [11].

Study results also suggest that, when planning psychotherapy, a subset of patients with severe psychopathology such as BPD or SoD might need particular assistance learning to deal with emotional distress by enhancing their capacity to activate mental representations of (new) attachment figures. For patients with fear of abandonment and under-regulation of emotion, the reparative attachment experience may involve therapeutic guidance and role modeling of ways to tolerate separation without experiencing extreme and unmanageable emotion states. For patients with fear of closeness and over-regulation of emotion, new attachment experiences and working models might need to specifically enhance their ability to benefit from intimacy while maintaining their emotional activation within tolerable limits without over-control. When both hyperactivation and deactivation secondary attachment strategies and both forms of emotion dysregulation co-occur, which appears particularly likely with comorbid BPD and SoD, treatment may need to address cyclically alternating states of apparently incompatible attachment fears and forms of emotion regulation.

### Limitations

Limitations of the study include that it was performed in a clinical setting with a sample limited to patients diagnosed with BPD, and/or SoD, although there was a comparison group of patients with other psychiatric disorders who had equally severe and persistent psychopathology. Inclusion of a broader range of clinical and non-clinical participants—children and adolescents as well as adults—would increase the generalizability of findings concerning emotion dysregulation, attachment, and psychopathology. The self-report measures used to assess emotion dysregulation and adult attachment have acceptable psychometric properties but have only preliminary validation for the diagnostic groups included in this study. Although assessment was embedded in clinical practice and diagnoses utilized clinical observations and documentation, study findings would be enhanced by direct observational measures or structured interviews in order to enhance the external validity of the findings.

## Conclusion

While BPD and SoD are often associated with insecure attachment, specific secondary attachment strategies of fear of abandonment (hyperactivation of the attachment system) or closeness (deactivation of the attachment system) appear to differentiate these clinical populations. Under-regulation of emotion, and fear of abandonment was particularly associated with BPD. Further, contrary to study hypotheses, fear of closeness also was associated with BPD, primarily when BPD was comorbid with SoD. SoD was associated with over-regulation of emotion and dismissive secondary attachment strategies, but was not consistently associated with fearful secondary attachment strategies (with fear of abandonment endorsed particularly infrequently) except when comorbid with BPD. The findings suggest that relatively distinct patterns of emotion dysregulation and secondary attachment strategies can be identified which are associated with specific patterns of psychopathology, and that these attachment/regulation impairments may also may occur together in adult patients who have complex comorbid psychopathology.

## Abbreviations

(BPD): Borderline personality disorder
(SoD): Somatoform disorders
(RSQ-NL): Relationship Style Questionnaire-NL
